# An analysis of rodent density patterns and its spatial–temporal correlations using geographically and temporally weighted regression in southeastern China

**DOI:** 10.3389/fvets.2025.1690571

**Published:** 2026-01-08

**Authors:** Mingyu Luo, Jinna Wang, Mingyong Tao, Hanran Ji, Guoqin Jiang, Qinmei Liu, Tianqi Li, Zhou Guan, Juan Hou, Zhenyu Gong

**Affiliations:** 1 Department of Communicable Disease Control and Prevention, Zhejiang Provincial Center for Disease Control and Prevention, Hangzhou, Zhejiang, China; 2 Key Lab of Vaccine, Prevention and Control of Infectious Disease of Zhejiang Province, Hangzhou, Zhejiang, China; 3 Department of Communicable Disease Control and Prevention, Hangzhou Center for Disease Control and Prevention, Hangzhou, Zhejiang, China; 4 Center for Global Public Health, Chinese Center for Disease Control and Prevention, Beijing, China; 5 Department of Vectors Prevention and Control, Shaoxing Prefectural Center for Disease Control and Prevention, Shaoxing, Zhejiang, China; 6 Department of Public Health Emergency Response, Zhejiang Provincial Center for Disease Control and Prevention, Hangzhou, Zhejiang, China

**Keywords:** One Health, public health, rodent density, GTWR, meteorological, vegetation

## Abstract

**Introduction:**

Rodents are significant vectors, harboring diverse pathogenic microorganisms which can spread a variety of infectious diseases. Surveillance on rodent density patterns should be prioritized as early warning indicators for prevention and control of infectious diseases. We aimed to analyze spatial and temporal heterogeneity of rodent density patterns in Zhejiang Province, and analyze the spatial and temporal correlations between rodent density, meteorological, land use and vegetation factors.

**Methods:**

Collect rodent surveillance data, meteorological factors, and vegetation factors in Zhejiang Province from 2019 to 2023. Analyze the temporal and spatial distribution of rodent density patterns. Employ the GTWR model to analyze the spatial and temporal correlations between rodent density, meteorological, land use and vegetation factors.

**Results:**

The rodent density in southern Zhejiang Province was higher than that in northern Zhejiang Province, and the species richness of wild rodents also exceeded that in northern Zhejiang Province. The effects of meteorological and vegetation factors on rodent density varied across geographical spatial distributions, which might primarily be related to the distribution range of rodent species in different habitat sites.

**Discussions:**

Meteorological and vegetation factors could influence the rodent density, particularly that of wild rodents, by offering a suitable environment for growth and development as well as food sources.

## 1 Introduction

The “One Health” concept guides national authorities and institutions in preventing vector-borne disease transmission by implementing comprehensive vector prevention and control measures (1). Rodents are significant vectors, harboring diverse pathogenic microorganisms within their body fluids and secretions, which can lead to the spread of a variety of infectious diseases such as hemorrhagic fever with renal syndrome (HFRS) and leptospirosis (2). In addition, emerging evidence from high-throughput sequencing and metaviromics research suggests that rodents are significant reservoirs of novel viruses or uncharacterized pathogens (3–5).

Rodent density is one of the determined factors for the transmission of rodent-borne diseases, an increase in rodent density may increase the risk of HFRS, plague and other rodent-borne diseases (6–8). The distribution characteristics of rodents can directly reflect their pattern of spatial clustering, and the spatial and temporal distribution of rodent density can affect the dynamics of natural foci of rodent-borne diseases (9). Currently, infectious disease surveillance and early-warning systems are receiving increasing attention in public health. Therefore, surveillance on vectors, such as rodent density patterns, should be prioritized as a key early-warning indicator (10).

The reproduction and distribution of rodents are directly or indirectly affected by meteorological, environmental, geographical and other factors (11). Specifically, meteorological factors (Temperature, precipitation, atmospheric pressure, humidity and others) and environmental factors (Land use classification, Vegetation Index and others) comprise multiple indicators. The interactions among factors with vectors and vector-borne diseases result in sophisticated causal networks (12). Previous studies centered on relatively confined zones or monitoring sites have revealed that the effects of meteorological factors on vectors and vector-borne diseases vary significantly across different geographical regions (13). Furthermore, previous studies on the relationships between meteorological factors, rodent density and rodent-borne diseases in China were mostly concentrated in the north or west regions (14–17), while the southeastern coastal areas remain understudied. This geographical bias represents a significant limitation in the current research landscape (18, 19). To more accurately describe relationships between meteorological and environmental factors, rodent density patterns and rodent-borne diseases, long-term temporal observation scales and broader spatial coverage should be considered.

Generally, due to spatiotemporal non-stationarity, the relationships or structures between factors undergo changes with variation in geographical locations or time series (12, 20). In recent years, the geographically and temporally weighted regression (GTWR) model has been implemented in the fields of infectious disease prevention and control and environmental health to identify influencing factors (21–25). The GTWR model comprehensively demonstrates the geographically discrete distribution and temporally fluctuation of data, and intuitively reflects the geographically and temporally aggregation and non-stationarity of data relationships. In the monitoring and early warning of infectious diseases, the GTWR model could effectively depict the spatial and temporal pattern of diseases, the associations between diseases and influence factors, and their geographical disparities and temporal (seasonal) dynamics. Thus, the GTWR model may also be used to analyze the associations between rodent density patterns and meteorological and environmental factors.

In this study, we aim to analyze spatial and temporal heterogeneity of rodent density patterns in Zhejiang Province, and analyze the spatial and temporal correlations between rodent density, meteorological, land use and vegetation factors.

## 2 Methods

### 2.1 Study design and setting

This is a retrospective cross-sectional study of rodent density patterns in Zhejiang Province from 2019 to 2023. The GTWR model was conducted to analyze the spatial and temporal correlations between rodent density, meteorological, land use and vegetation factors.

### 2.2 Study area

Zhejiang Province is located on the southeastern coast of China (27°02’N to 31°11’N and 118°01′E to 123°10′E). It experiences a subtropical monsoon climate characterized by distinct seasonal variations, including a warm and humid environment with a hot summer, which create suitable conditions for the growth and reproduction of rodents and other vectors (26). Consequently, surveillance on rodent-borne diseases is continuously valued in infectious disease prevention and control in Zhejiang Province.

### 2.3 Data source and collection

Rodent density and species assemblage data was obtained from vector surveillance database of Zhejiang Provincial Center for Disease Control and Prevention (CDC). The rodent surveillance procedures and capture methods was conducted in accordance with the monitoring plan released by the China CDC, and National Standard: Surveillance methods for vector density-Rodent (GB/T 23798-2009). In total of 90 county-level administrative divisions in Zhejiang Province, each county has set three types of surveillance habitats: urban residential areas (e.g., urban villages, urban and rural fringe areas, urban community, etc.), rural residential areas (e.g., natural village in rural areas, the crop land, the mountainous region and shrubbery, etc.), and the key industries (e.g., Catering places, food production and sales sites, construction sites, slaughter houses, brewing plants, etc.).

Among three trapping methods (trap, cage, glue board), each habitat used at least two methods to trap rodents during the middle 10 days of January, March, May, July, September, and November. The medium rodent traps, rodent cages or rodent glue boards were distributed every 15 m^2^ indoors or every 5 m along the wall root in rooms over 100 m^2^. In the crop land, the mountainous region, the shrubbery and other rural areas, the rodent traps or rodent cages were placed every 5 m, with a row spacing not less than 50 m. At least 200 rodent traps were placed in each monitoring habitat, and a total of 600 rodent traps were placed in each district or county each monitoring. All the traps were placed at dusk and taken back in the morning.

The trapping locations were identified based on prior field experience and observations of local environmental indicators of rodent activity. The surveillance should not be carried out in the same location within 3 months, and the distance between the locations selected in different months should be greater than 250 m.

Calculate rodent density via rodent which were trapped:


$$\text{Rodent Density}=\frac{\text{Number of trapped rodents}}{\text{Number of effective traps},\text{cages},\text{or glue boards}}\times 100$$

### 2.4 Meteorological factors

Temperature (including mean temperature, maximum temperature, minimum temperature) and precipitation database were provided by National Tibetan Plateau/Third Pole Environment Data Center^1^ (27–30); Other meteorological factors including gradient surface temperature, atmospheric pressure, relative humidity, sunshine duration, and wind speed were obtained from Monthly spatial interpolation dataset of meteorological elements in China, which was provided by Resource and Environmental Science Data Registration and Publishing System, Institute of Geographic Sciences and Natural Resources Research (31).

### 2.5 Vegetation factors

Normalized Difference Vegetation Index (NDVI) was obtained from China regional 250 m normalized difference vegetation index data set (2000–2023), which was provided by National Tibetan Plateau/Third Pole Environment Data Center (see text footnote 1) (32).

Land cover classification was obtained from “The 30 m annual land cover datasets and its dynamics in China from 1985 to 2023,” which was published by Prof. Jie Yang, and Prof. Xin Huang in Wuhan University (33). Calculated proportion of four kinds of land (crop land, forest, shrub, grassland) in the local county-level administrative division area.

### 2.6 Analysis and statistics

Excel 2019 was used to construct the database and to produce all descriptive tables and figures. Spatial and temporal analysis and associated visualizations were carried out in ArcGIS 10.8 (Esri Inc., Redlands, CA, United States). The 2024 administrative-boundary vector data (Map Examination Number: GS (2024)0650, Coordinate system: GCS_WGS_1984), released by the National Geomatics Center of China, were adopted. Five-fold cross-validation was performed in R 4.4.2, and model performance evaluation was evaluated using the caret package.

The specific procedures and analyses were as follows: 1. Global spatial autocorrelation was assessed using Moran’s I Index to quantify the spatial aggregation patterns and degree of autocorrelation of the specific factors. 2. Multicollinearity among variables was tested using the Variance Inflation Factors (VIF) to screen suitable variables for the construction of GTWR model. 3. Ordinary Least Squares (OLS), Geographically Weighted Regression (GWR), and GTWR models were established. Their performances were compared based on the Akaike Information Criterion (AICc) value to evaluate their goodness of fit. 4. Five-fold cross-validation was performed, predictive performances of GWR and GTWR models were compared based on the R^2^(cv) (Mean coefficient of determination from 5-fold cross-validation); prediction accuracy of both models was compared based on the Root Mean Square Error (RMSE) and Mean Absolute Error (MAE); model stability of both models was compared based on the Coefficient of Variation of RMSE [CV(RMSE)] and Coefficient of Variation of R^2^ [CV(R^2^)].

GTWR model was constructed to analyze relationships between rodent density, meteorological factors and vegetation factors, the coefficient values were analyzed with “0” serving as the boundary to distinguish the positive and negative coefficient; Coefficients with positive values indicated a positive correlation between the factors and rodent density; Conversely, coefficients with negative values indicated a negative correlation between the factors and rodent density. Visualizations were produced to display geographical distribution of rodent density, rodent species assemblage, meteorological factors and vegetation factors. Visualizations were produced to display the coefficients between rodent density and meteorological and vegetation factors, and their temporal–spatial distribution dynamics.

## 3 Results

### 3.1 Temporal and spatial distribution of rodent density and rodent species assemblage

From January 2019 through November 2023, a total of 1,826,275 effective traps (including rodent traps/cages/glue boards) were set in all 90 counties of Zhejiang Province; 8,908 rodents were trapped, and the rodent density was 0.48 per 100 traps/cages/glue boards. Among 8.908 trapped rodents, there were 6,100 commensal rodents (68.48%), and 2,808 wild rodents (31.52%); *Rattus norvegicus* accounted for the highest proportion (37.23%), with a total of 3,317; and *Suncus murinus* accounted for the second highest proportion (21.65%), with a total of 1,929.

**Figure 1:**
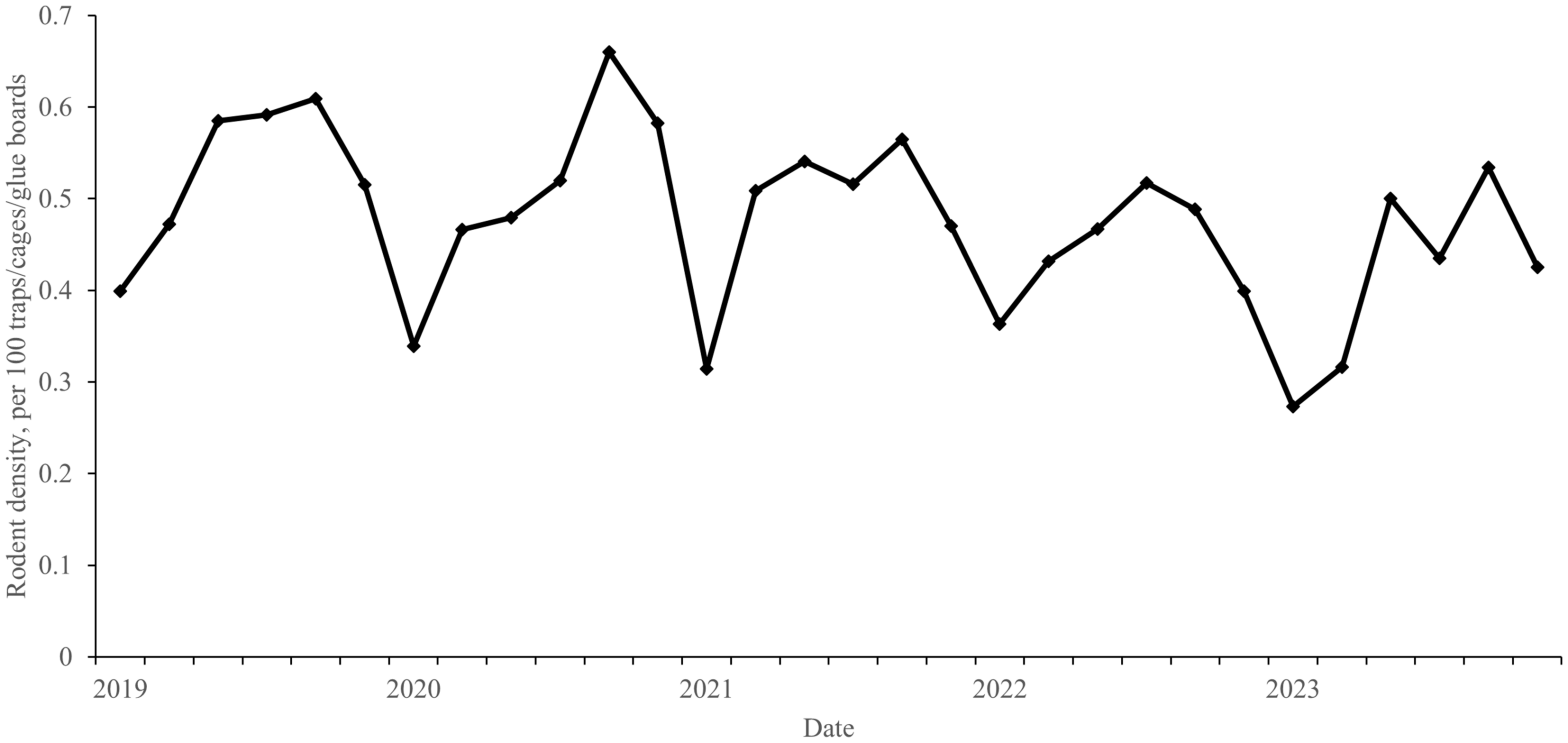
Seasonal fluctuation of rodent density in Zhejiang Province from January 2019 through November 2023.

**Figure 2:**
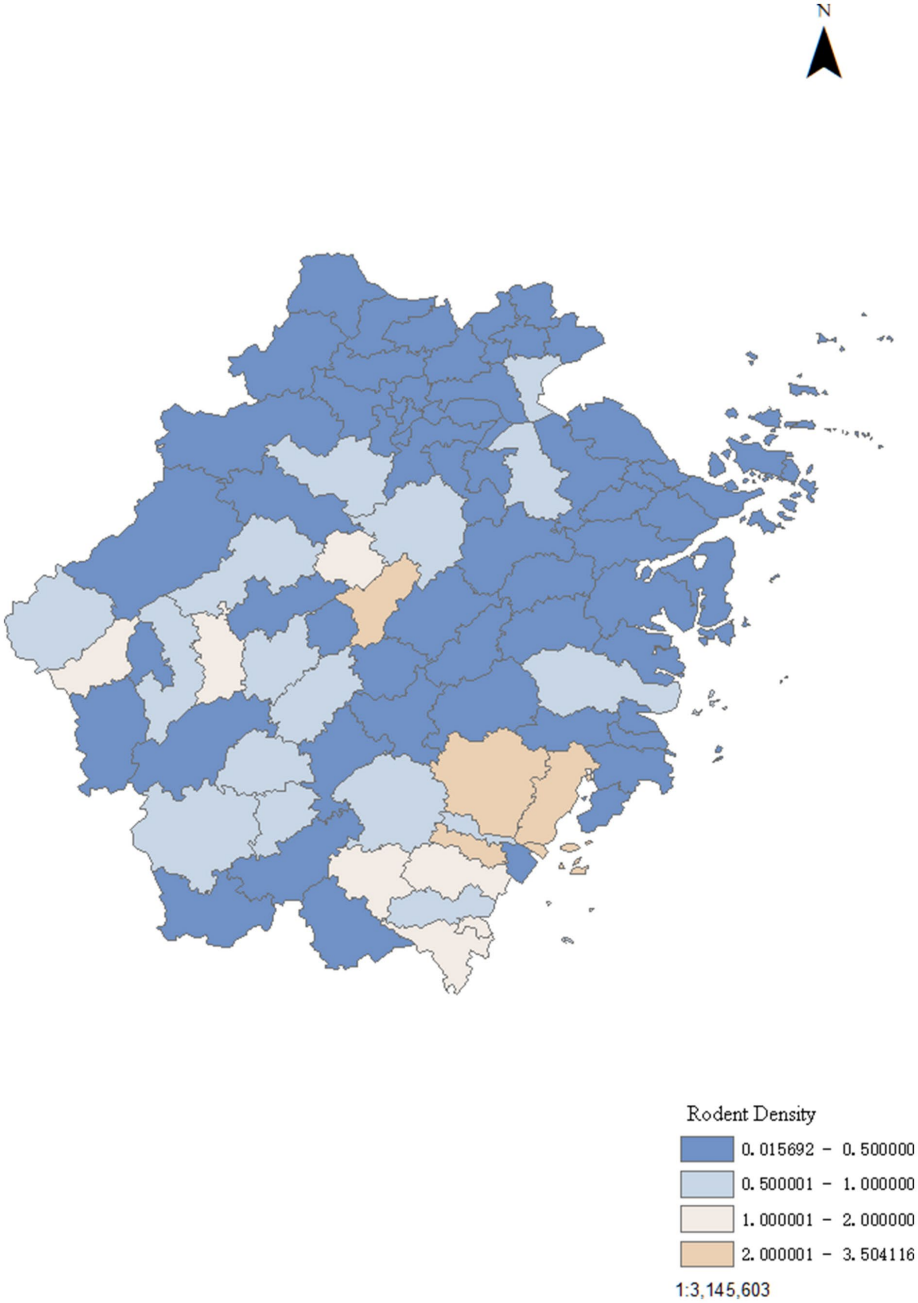
Geographical distribution of mean rodent density in Zhejiang Province from January 2019 through November 2023.

The rodent density exhibited seasonal fluctuation, typically characterized by a bimodal pattern with double peaks in the spring (May) and autumn (September), however, this bimodal deviated from the norm in 2020 and 2022 (Figure 1). Over the past 5 years, a total of 12 counties in Zhejiang Province exhibited average rodent density ≥1 per 100 traps/cages/glue boards. The rodent density showed a trend of gradual increase from north to south of Zhejiang Province, and counties with higher rodent density were predominantly concentrated in the southeast coastal areas (Figure 2).

**Figure 3:**
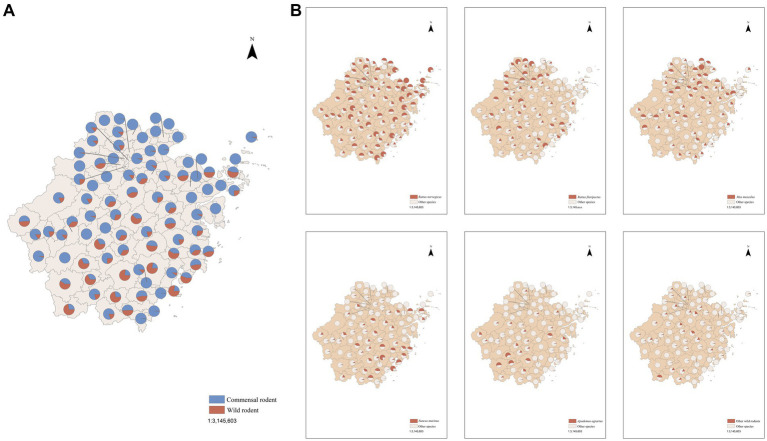
Geographical distribution of rodent species assemblage in Zhejiang Province from January 2019 through November 2023. **(A)** Geographical distribution of composition of commensal rodent and wild rodent in Zhejiang Province from January 2019 through November 2023. **(B)** Geographical distribution of proportion of each rodent species in Zhejiang Province from January 2019 through November 2023; Other wild rodents referred to wild species other than *Suncus murinus* and *Apodemus agrarius*, including *Niviventer confucianus*, *Berylmys bowersi*, etc.

In terms of rodent species assemblage, counties with a higher proportion of commensal rodents, such as *Rattus norvegicus*, were mainly distributed in the northern and western regions of Zhejiang Province. Conversely, counties with a higher proportion of wild rodents, such as *Suncus murinus* and *Apodemus agrarius* were mainly distributed in the central and southern regions. The proportion of wild rodents roughly showed an increasing trend from central and western areas to southeastern area (Figures 3A,B).

### 3.2 Spatial analysis

#### 3.2.1 Construction of GTWR model

Spatial autocorrelation analysis showed that there was spatial aggregation in the geographical distribution of rodent density across Zhejiang Province (*Moran’s Index* = 0.3451, *p* < 0.001).

A total of 14 variables, encompassing meteorological factors and vegetation factors, were incorporated into the multicollinearity test, and four variables exhibiting high multicollinearity were excluded: Proportion of forest in the local county-level administrative division area (*VIF* = 14.0867), monthly minimum temperature (*VIF* = 182.8925), monthly maximum temperature (*VIF* = 105.1255), Monthly average Gradient Surface Temperature (*VIF* = 127.1132).

The comparison of model fit indices revealed the superiority of the GTWR model: The AICc value of the GTWR model was lower than those of the OLS and GWR models, while the R^2^ and adjusted R^2^ of the GTWR model were higher than the OLS and GWR models, which showed that the GTWR model could better explain the effects of each variable on rodent density (Table 1).

**Table 1:** Values of AICc, R^2^ and adjusted R^2^ of OLS, GWR, and GTWR models.

Model	AICc	R^2^	Adjusted R^2^
OLS	6425.2569	0.0829	0.0794
GWR	5225.7700	0.4592	0.4572
GTWR	5194.9900	0.4933	0.4915

AICc, Corrected Akaike Information Criterion; OLS, Ordinary Least Squares; GRW, Geographically Weighted Regression; GTWR, Geographically and Temporally Weighted Regression; R^2^ and Adjusted R^2^ were calculated on the entire training set.

Five-fold cross-validation demonstrated the superior predictive performance of the GTWR model compared to the GWR model. The GTWR model achieved a mean R^2^(cv) of 0.5089, representing an 8.4% improvement over the GWR model. In terms of prediction accuracy, the GTWR model exhibited lower error rates, with RMSE and MAE values of 0.5852 and 0.3645, respectively, compared to 0.6048 and 0.3764 for the GWR model. Regarding model stability, both models showed good consistency across validation folds. The GTWR model demonstrated excellent stability in explanatory power, with a lower R^2^ coefficient of variation. Both models maintained acceptable error stability, with CV(RMSE) values below 15% (Table 2).

**Table 2:** Results of cross-validation of GWR and GTWR models.

Model	R^2^(cv)	RMSE	MAE	CV(RMSE)	CV(R^2^)
GWR	0.4693	0.6048	0.3764	11.76%	9.35%
GTWR	0.5089	0.5852	0.3645	12.00%	8.47%

R^2^(cv), Mean coefficient of determination from 5-fold cross-validation; RMSE, Root Mean Square Error; MAE, Mean Absolute Error; CV(RMSE), Coefficient of Variation of RMSE; CV(R^2^), Coefficient of Variation of R^2^.

#### 3.2.2 Spatial distribution of coefficients

**Figure 4:**
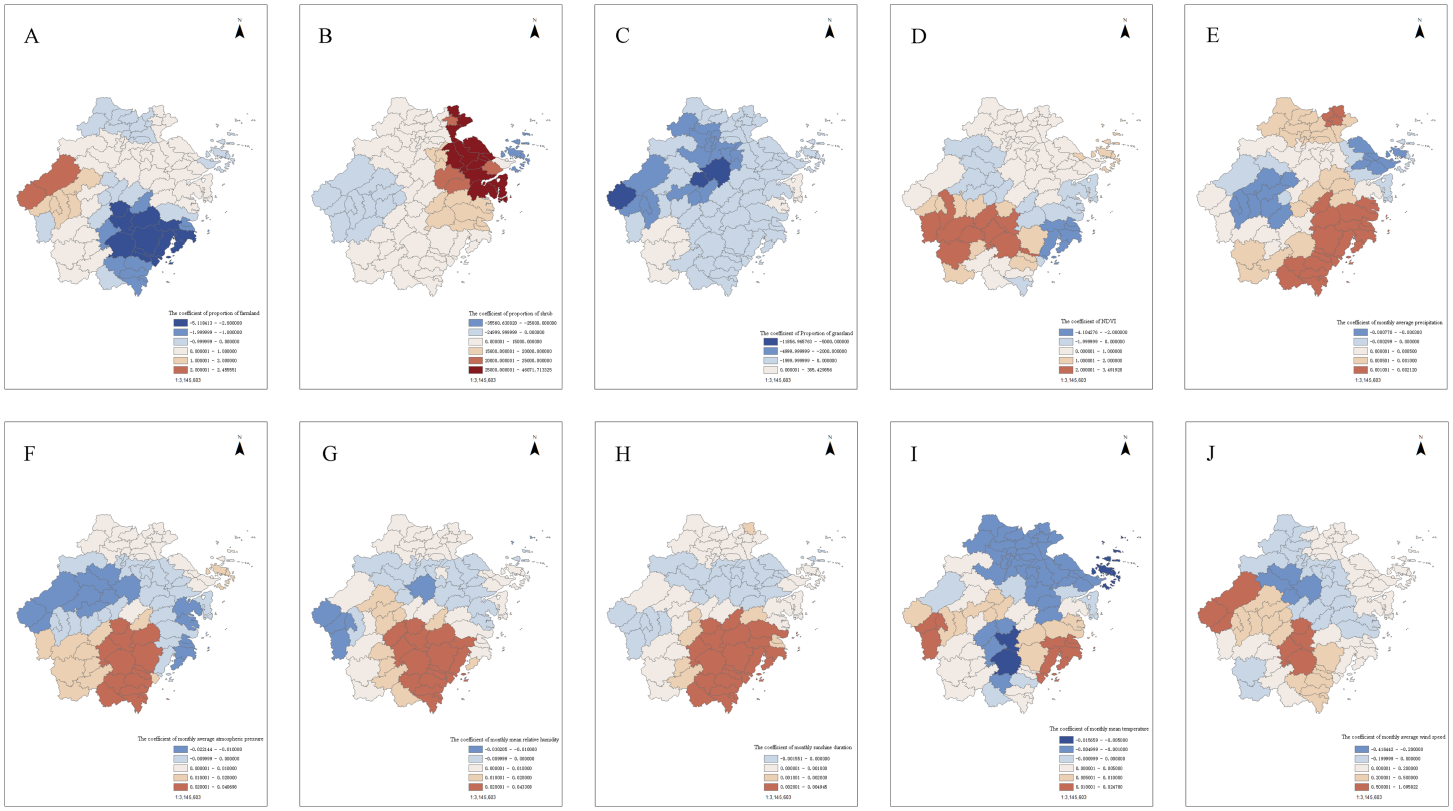
Geographical distribution of coefficients of each variable in Zhejiang Province (January 2023). Geographical distribution of coefficients of **(A)** proportion of crop land, **(B)** proportion of shrub, **(C)** proportion of grassland, **(D)** NDVI, **(E)** monthly average precipitation, **(F)** monthly average atmospheric pressure, **(G)** monthly mean relative humidity, **(H)** monthly sunshine duration, **(I)** monthly mean temperature, **(J)** monthly average wind speed.

Draw spatial and temporal distribution maps of each coefficient based on the results of the GTWR model, and the patterns of spatial distribution demonstrated variables’ spatially heterogeneous effects on rodent density. The spatial distribution of January 2023 was presented in Figure 4. The coefficient values of different time periods had some differences, however, the spatial distribution patterns roughly remained consistent (Geographical distribution of mean value of each variable was also provided in Supplementary Figure S1).

From January to November, except for a few counties in the northern areas and northeastern sectors, the coefficient of proportion of crop land showed a declining trend along a northwest-to-southeast spatial gradient across Zhejiang Province. The proportion of crop land showed a positive correlation with rodent density in the northwestern areas, and a negative correlation in the southeastern areas.

From January to November, except for a few counties in the northeast areas, the coefficient of proportion of shrub showed an increasing trend from northeast to southwest. The proportion of shrubs was positively correlated with rodent density in most areas, except in a small number of counties in the northeast and west areas, where a negative correlation was observed.

From January to November, the coefficient of proportion of grassland showed an increasing trend from north to south. The proportion of grassland was negatively correlated with rodent density in most areas. However, in a small number of counties within the southwestern and eastern areas, a positive correlation was observed.

From January to November, the coefficient of NDVI showed a gradually increasing trend from north to south. The NDVI was positively correlated with rodent density in most areas. In a few counties in the central and western areas, as well as in some counties in the southeastern areas, which showed a banded distribution, a negative correlation between NDVI and rodent density was observed.

From January to November, the coefficient of monthly average precipitation showed an increasing trend from central-western areas to southeast areas, except for a few counties in the northeast area. The monthly average precipitation was positively correlated with rodent density in most areas, except for a few counties in central-western and northeastern areas, where a negative correlation was observed.

From January to November, the spatial distribution patterns of the coefficient of monthly average atmospheric pressure showed an increasing trend from the central-western area to the north and southeast areas. In the central area, a large band-shaped area from west to east, monthly average atmospheric pressure showed a negative correlation with rodent density, while in a few counties in the north area and across a large expanse of the southern area, a positive correlation was found.

The spatial distribution patterns of the coefficient of monthly mean relative humidity, and monthly sunshine duration exhibited similarities, with a general increasing trend from the central-western to the southeastern areas, except for some counties in the northern area. In the central areas, a band-shaped area from west to east (except for a few countries in the northeastern corner), and a few counties in the western corner, these two variables demonstrated a negative correlation with rodent density; while across a large expanse of the southern area and a few counties in the northern area, they demonstrated a positive correlation. However, a distinct temporal variation is observed for monthly mean relative humidity: from January to November, the number of counties with negative correlation were increased in the northern area. In contrast, the spatial distribution of the positive or negative correlation between rodent density and the other two variables remained relatively stable over time.

Except for a few numbers of counties, from January to November, the coefficient of monthly mean temperature showed an increasing trend from the northeast to the southeast and southwest. Approximately half of the whole Zhejiang Province in northeast and central-southern areas, monthly mean temperature was negatively correlated with rodent density, while in the southeast and southwest areas, a positive correlation was observed. The spatial distribution patterns of the coefficient of these three variables also had a degree of overlap and similarity with the spatial distribution pattern of the coefficient of monthly average precipitation, but the areas showed negative correlation with rodent density in the central-west and northeast formed contiguous areas.

From January to November, the coefficient of monthly average wind speed showed an increasing trend from central and northwestern areas to northeastern and southwestern areas. In the diagonally distributed zones of the central and northwestern areas, as well as in isolated counties in the southwestern areas, the monthly average wind speed was negatively correlated with rodent density; Conversely, in other areas, a positive correlation was observed.

## 4 Discussion

The spatial and temporal distribution of rodent density patterns profoundly influence the maintenance of the natural foci of rodent-borne diseases. Surveillance has consistently identified rodent density as a fundamental determinant of disease incidence, as clearly evidenced by its correlation with HFRS. Moreover, higher rodent density is positively associated with an increased risk of ectoparasite infestation, amplifying the potential for pathogen transmission (34).

The results of this study showed that rodent density not only fluctuates in a non-linear manner across temporal scale, but also displays aggregation across geographical areas. The rodent density in Zhejiang Province typically peaked in May and September. These months are associated with the spring and autumn breeding seasons of rodents, when the population is increasing. The results also showed that rodent density demonstrated a gradual increase from the northern to the southern part of Zhejiang Province, with counties exhibiting higher rodent density predominantly concentrated in the southeastern coastal areas.

Results of this study showed that counties with a relatively higher proportion of commensal rodents were primarily situated in the northern and western areas, while counties with a higher proportion of wild rodents were mainly distributed in the central and southern areas. According to field experience of staff from local Centers for Disease Control and Prevention (CDC), this north-to-south increase in wild rodent prevalence was accompanied by greater species diversity. Wild rodent species such as *Leopoldamys edwardsi* and *Berylmys bowersi*, which were rare across the province, showed a greater likelihood of occurrence in the south.

The southern part of Zhejiang Province is more mountainous, whereas the northern part is relatively flat. Additionally, the northern area has a more thriving economy and an accelerating process of urban construction. The intervention of human activities has a complex dua effect on rodent community. On one hand, as urban construction expands, the original ecological environment has been altered and the habitat sites available for rodents have been reduced. On the other hand, this situation also leads to the migration of rodent habitat sites and the concentration of rodent density in local areas. One significant consequence of this is the heightened probability of interaction and intermingling between different rodent species, particularly between commensal rodents and wild rodents, which facilitates the exchange and aggregation of pathogens among rodents. In addition, the human living environment may further intensify the existing epidemic areas. This results in an increased risk of humans contact with rodents that carry pathogens.

Zhejiang Province experiences four distinct seasons. The data in this study, spanning from January to November, encompassed the months exhibiting the highest and lowest values of various meteorological and vegetation factors. However, throughout the year, the geographical distribution of the regression coefficients for these factors on rodent density showed no significant changes. The climate in Zhejiang Province is relatively mild, and extreme weather events are rare. Furthermore, Zhejiang Province has a rich and relatively stable ecosystem when compared to the desert ecosystem with scarce resources. The seasonal variations of vegetation factors are more restrained and less responsive to climate change. In addition, for most meteorological and vegetation factors included in this study, the geographical distribution of their mean values was inconsistent with that of their regression coefficients.

In general, the results demonstrated that meteorological and vegetation factors have an impact on rodent density. This was mainly elucidated by the intricate non-linear relationship between the geographical distribution of these factors and the fluctuations in rodent density.

The results showed that coefficient of monthly mean temperature showed an increasing trend from northeastern area to southeastern and southwestern areas. The areas where the monthly mean temperature showed a negative correlation and a positive correlation with rodent density were roughly equal in proportion. Notably, even in areas where the temperature showed a negative correlation with rodent density, the absolute regression coefficient values were relatively small.

Based on comprehensive observations of the global climate system and numerous key indicators, the trend of climate warming persists (35). It is generally considered that climate warming can expand the spatial and temporal scope of the environment suitable for the growth and reproduction of rodents. This expansion contributes to a wider distributional range across bioclimatic zones and a prolonged seasonal activity (36). Previous studies have primarily indicated a non-linear relationship between temperature and rodent activity (13). Specifically, rodent density requires a suitable temperature range: Temperatures between about 20 and 30 °C are favorable for rodent density; in contrast, when temperatures exceed 30 °C or fall below 10 °C, their activity intensity decreases significantly. Moreover, the effect of temperature on rodent density exhibits a notable time lag (19, 37). According to the distribution of average temperature, there is a certain degree of overlap between the areas where the temperature positively correlates with the rodent density and areas with higher temperature. Additionally, even in the areas with higher average temperatures in Zhejiang Province, the temperature range is generally suitable for the growth and reproduction of rodents.

The impact of temperature on rodent density may intertwine with factors like diverse habitats and rodent species distribution. A previous study in Shanghai, also in the Yangtze River Delta region, found that farmland rodent density mostly had a negative correlation with monthly average temperature, except for a weak positive correlation in January (38). Research in northern China’s desert areas revealed that different rodent species have varying heat tolerances (39). We speculated that similar features also exist in the southern areas, which have abundant ecological resources and adaptation to the survival of vector organisms. Therefore, there are differences in the relationship between temperature and rodent density in different local areas with different dominant rodent species.

The geographical distributions of the regression coefficients for several meteorological factors, including monthly average atmospheric pressure, monthly average wind speed, monthly average precipitation, monthly mean relative humidity, and monthly sunshine duration, showed notable similarities. In particular, the coefficients for monthly average precipitation, monthly mean relative humidity, and monthly sunshine duration exhibited a general increasing trend from the central-western areas to the southeast. In the central area, there was an approximate horizontal banded area where these three meteorological factors showed a negative correlation with rodent density. Monthly average atmospheric pressure was characterized by a broader banded area; Monthly average wind speed was distributed diagonally across the central and northwestern areas, as well as in isolated counties in the southwestern areas; Monthly precipitation was characterized by two separate areas in the central-western and northeastern areas; Monthly mean relative humidity and monthly sunshine duration were almost identical in distribution, both were present in the central-western and northeastern areas (except for the northeastern corner), though they did not form a continuous banded area. Meanwhile, in the northern area and the large areas of the south, these meteorological factors all showed a positive correlation with rodent density.

Previous studies have demonstrated that moderate precipitation and humidity can boost rodent density. In arid areas, the increase in precipitation can promote the rodent density, while in humid areas, the increase in precipitation may have the opposite effect and can decrease the rodent density. It is widely accepted that atmospheric pressure primarily affects animals’ activity through its impact on precipitation (40). The decrease in atmospheric pressure is associated with the increase in precipitation, while the increase in atmospheric pressure is related to the increase in sunshine duration. The photoperiod is considered to be an important environmental signal for the seasonal reproduction of mammals. The increase in sunshine duration can stimulate the testicular enlargement of male wild rodents, thereby enhancing their reproductive capacity (41).

In Zhejiang Province, the average wind speeds are relatively low, except in some eastern coastal counties. Moderate wind speeds can enhance plant photosynthesis and pollen transmission by promoting gas flow, which in turn promotes plant growth and provides more food for wild rodents, thus facilitating their growth and reproduction.

The areas where these meteorological factors promote the rodent density largely overlap with the areas where wild rodents were more concentrated, suggesting that in areas with milder climates, such as Zhejiang Province, different meteorological factors acted synergistically to promote the growth of wild rodents in outdoor activities.

NDVI is an important parameter for detecting vegetation growth status and vegetation cover status. It presents the biomass of plants on a macro scale and is the most widely used vegetation index. Vegetation can not only provide shelter and habitat for small animals such as rodents, but more importantly, it can be used as a food source parameter for small animals by better reflecting the richness and yield of vegetation such as crops (36, 42). In most areas, NDVI was positively correlated with rodent density. However, in a few counties in the central-western and southern areas, a negative correlation between NDVI and rodent density was observed.

This study encompassed four variables related to vegetation across different land types, including crop land, forest, shrub, grassland, and calculated the proportion of each land type. Finally, the proportion of crop land, shrub, and grass was incorporated into the GTWR model. Based on the distribution of the regression coefficients, the areas where the proportion of crop land showed a positive correlation with rodent density largely corresponded to areas with a high proportion of commensal rodents. This indicated that crop land primarily serves as a food resource for commensal rodents. The areas where the proportion of shrub showed a positive correlation with rodent density essentially encompassed the areas with a high proportion of wild rodents. The food sources for wild rodents are mainly the stems and leaves of herbaceous plants and dwarf shrubs. In most areas, the proportion of grassland showed a negative correlation with rodent density, which might be due to the absence of rodent species in Zhejiang Province that are dependent on grassland environments, such as *Ochotona curzoniae*, *Eospalax fontanierii*, and *Meriones unguiculatus*.

In summary, the rodent density in southern Zhejiang Province was higher than that in northern Zhejiang Province, and the species richness of wild rodents also exceeded that in northern Zhejiang Province. The effects of meteorological and vegetation factors on rodent density varied across geographical spatial distributions, which might primarily be related to the distribution range of rodent species in different habitat sites. Meteorological and vegetation factors could influence the rodent density, particularly that of wild rodents, by offering a suitable environment for growth and development as well as food sources. However, this study had certain limitations. Although all monitoring sites followed the authoritative monitoring procedures and capture methods, there was no entirely uniform rodent capture method, which might have affected the representativeness of rodent density. There are other factors not included in the model that could influence rodent density. In addition to other natural factors (e.g., topography, soil composition), human activities, such as urbanization, and vector control and prevention strategies should also be incorporated into future models. Another limitation of the study is that the GTWR model only applies to total density. The observations on species came from descriptive analyses rather than the GTWR, which explains global abundance patterns but not community composition.

## Data Availability

The raw data supporting the conclusions of this article will be made available by the authors, without undue reservation.

## Footnotes

1
http://data.tpdc.ac.cn

## Supplementary material

Supplementary Figure S1 Geographical distribution of mean value of each variable in Zhejiang Province from January 2019 through November 2023.
